# Elastography of the Male Pelvic Region—Perspectives on Malignant Lesions

**DOI:** 10.3390/diagnostics14121218

**Published:** 2024-06-08

**Authors:** Rute Santos, Martina Kastrup Loft, Malene Roland Vils Pedersen

**Affiliations:** 1 Medical Imaging and Radiotherapy Department, Coimbra Health School, Polytechnic University of Coimbra, 3045-093 Coimbra, Portugal; 2 H&TRC—Health & Technology Research Center, Coimbra Health School, Polytechnic University of Coimbra, 3045-093 Coimbra, Portugal; 3 CIPER-UC, University of Coimbra, 3004-531 Coimbra, Portugal; 4 Department of Radiology, University Hospital of Southern Denmark, Vejle Hospital, Beriderbakken 4, 7100 Vejle, Denmark; maloft@rm.dk (M.K.L.); malene.roland.vils.pedersen@rsyd.dk (M.R.V.P.); 5 Department of Radiology, University Hospital of Southern Denmark, Kolding Hospital, Sygehusvej 24, 6000 Kolding, Denmark; 6 Department of Regional Health, Faculty of Health, University of Southern Denmark, Campusvej 55, 5230 Odense, Denmark; 7 Discipline of Medical Imaging & Radiation Therapy, School of Medicine, University College Cork, T12 AK54 Cork, Ireland

**Keywords:** shear wave elastography, pelvis, ultrasound

## Abstract

Ultrasound elastography is widely used to assess tissue stiffness for lesion characterization, including differentiation between benign and malignant lesions. This study focuses on the use of elastography in the male pelvis, including the prostate, testicles, and rectum, by comparing elastography types (shear wave and strain). This article provides a summary of the existing literature on the use of elastography in the male pelvic region and outlines the clinical perspective. Ultrasound elastography is a good technique for evaluating and monitoring lesions in the male pelvic region.

## 1. Introduction

Elastography is a non-invasive technique that allows for the characterisation of the mechanical properties of tissues, aiming to determine the respective Young’ modulus or the amount of deformation that the tissue undergoes when a load is applied [1]. This technique has been evolving rapidly, demonstrating great potential not only in the diagnosis of diseases characterised by alterations in tissue stiffness but also for the physiological and morphological study of different structures/tissues [2].

Elastography started as a qualitative and/or semi-quantitative technique, but has evolved into a quantitative approach by offering elasticity maps with the use of conventional ultrasonography equipment in real time, due to software developments [3,4]. Ultrasound (US) elastography was described for the first time by Ophir et al. in 1991 [5] and, later on, it evolved into a real-time imaging tool [6]. It may be defined as a dynamic technique developed to provide an estimated value of the elasticity/hardness of the tissue by measuring the degree of its distortion when subjected to an external force [7]. Elastography is a complementary technique to B-mode US, offering high diagnostic sensitivity regarding detection and assessment of the nature and structure of pathologic alterations in the body [8,9].

This first studies employing US elastography were carried out for the diagnosis of breast, thyroid, and prostate neoplasms [10]. Later on, advances in this method made it possible to study deeper structures, such as the liver and pancreas [7,11,12].

The type of elastography used depends on the method of stress application and its aims. The types of elastography include compression elastography, shear wave elastography, and transient elastography. Each one has advantages, artefacts, limitations, and specific clinical applications [13]. However, all types of elastography operate following three steps: stress or distortion applied to the region of interest (ROI), the tissue’s response (strain), and processing the distortion [14]. The differences between different elastography methods also reside on how the distortion is applied to the tissue and on the type of force that is applied.

### 1.1. Physical Principles of Elastography

Assuming that tissues are elastic (i.e., they return to their initial shape after undergoing deformation), isotropic (their elastic modulus does not depend on the orientation of the tissue), incompressible (no volumetric variations when deformed), and homogeneous, there exists four fundamental moduli that may be associated with each other: the modulus of elasticity or Young’s modulus, shear modulus, volumetric modulus, and Poisson’s ratio [1,15].

The elastic modulus or Young’s modulus is a mechanical parameter that is proportional to the rigidity of a solid structure when subjected to external tension or compression [10,16]. The shear modulus is based on the sliding of planes parallel to each other when forces are applied in parallel [16]. The volumetric modulus measures the tendency of a tissue to deform in all directions when applying a multidirectional force [1,9,16]. Poisson’s ratio measures the transverse deformation of a tissue when a longitudinal force is applied [15,16].

The basic principle of elastography is that stress applied to a tissue causes changes within it, which depends on its elastic properties. Elastography evaluates tissues elasticity by taking into account its deformation when a force is applied [9,17,18].

Since its emergence, different generations of elastography have been developed, depending on the type of stress application and the method used to detect tissue displacement and to obtain the image. However, all types of elastography use force and measure the deformation produced by this force on the tissue [9,17,18].

### 1.2. Elastography Artefacts

Despite great improvements in elastography, there are some important artefacts associated with this technique [2,19]. For example, the force–strain relationship is non-linear and time-dependent, the elasticity varies spatially and with direction, the contours and the structures of tissues can alter the relationship between the shear wave velocity and the shear modulus, and tissues can be mechanically discontinuous due to anatomical features, such as tumours or scars [2]. It is important to be familiar with the pitfalls and artefacts of US elastography for correct interpretation of the elastograms.

### 1.3. Methods for Force Application

Two types of elastography can be considered regarding the method used to apply force: quasi-static and dynamic.

#### 1.3.1. Quasi-Static Elastography (Strain)

In quasi-static elastography (QSE), stress is applied to the tissue using external vibration generated by a transducer [2,14,16]. The obtained images are semi-quantitative and do not directly describe the elasticity of the tissue since the amount of tension produced within the tissue is unknown. However, ROIs can be drawn in the area under study and in a reference region in order to calculate the ratio and obtain a semi-quantitative analysis [2,14]. Along with the absence of true quantification, a major limitation of QSE is the lack of control over the applied force. Moreover, the use of operator-imposed pressure limits the study to surface structures [16].

#### 1.3.2. Dynamic Elastography

In dynamic elastography, the force or source of stress is generated by the US probe [14]. The force applied can be a time-varying force, a short transient mechanical force, or an oscillatory force with a fixed frequency [16]. This method of US elastography has the advantages of being quantitative and of not depending on the operator or an external actuator to produce the stress [2].

### 1.4. Elastography Techniques

The main elastography techniques used are strain elastography, acoustic radiation force impulse elastography, transient elastography, and shear wave elastography. However, we focused on strain and shear wave elastography because they are the main techniques applied to male pelvic structures.

#### 1.4.1. Strain or Compression Elastography

Strain elastography, also described as compression elastography, is based on the quasi-static method and involves qualitative or semi-quantitative analysis [13,16]. The quantified parameter is strain, which is produced by repeatedly applying manual pressure to the tissue under investigation [13,20].

The difference in the echo produced by the pressure/strain is calculated (the modulus of elasticity = stress/strain), measuring the relative strain of one area compared to that of another. The results are represented by a colour-coded strain distribution map (elastogram), which is often superimposed over the conventional B-mode image or displayed side by side [13,20]. The elastogram is adjustable by the user, and this technique can draw the calculation area as a relatively free shape [13,20]. Most compression elastography equipment provides visual information about the applied pressure directly on the screen during examination [20].

#### 1.4.2. Shear Wave Elastography

Shear wave elastography is a dynamic method based on measuring the propagation velocity distribution of the directional shear wave produced by an US pulse [13,18]. The velocity of the shear wave can be measured and used to evaluate tissue elasticity using Young’s modulus (E), which is calculated using the formula E = 3rV2 (where E = Young’s modulus, V = shear wave velocity, and r = material density) [13]. This technique provides both qualitative elastograms and quantitative measurements, which are presented in quantitative maps with units in kPa (stiffness) or in centimetres per second (shear wave velocity) [13,18]. Shear wave elastography has a depth limitation, and only limited ROI shapes are available for the quantitative measurement of elasticity [13].

The aim of this literature review was to summarize the role of elastography in characterising focal lesions in the male pelvic region.

## 2. Rectal

### 2.1. Introduction

Rectal cancer is common, with more than 700,000 new cases per year worldwide [21]. Radiological imaging is an important part of preoperative diagnosis and may influence the treatment decision. Magnetic resonance imaging (MRI) is the recommended modality according to international guidelines [22,23]; however, MRI tends to overstage early rectal cancer (ERC). Therefore, adding Endorectal Ultrasound (ERUS) is recommended due to improved visualization of the layers of the rectal wall [24].

### 2.2. Rectal Anatomy and the Staging of Rectal Cancer

Conducting a successful endorectal ultrasound examination relies on a thorough knowledge of rectal anatomy. The rectum is the end of the large intestine (15 cm) and is typically divided into three parts, the upper, middle, and lower parts located 15–10, 10–5, and 5–0 cm from the anal verge, respectively. The wall consists of several layers. US imaging enables visualization of five echo layers of the rectum. First, a hyperechoic layer represents the interface between the transducer and the superficial mucosal layer. This is followed by a hypoechoic layer representing the mucosa and muscularis mucosa. A second hyperechoic layer represents the submucosa, followed by another hypoechoic layer representing the muscularis propria. Lastly, a hyperechoic layer represents the interface between the rectal wall and the surrounding mesorectal fat or the serosa [24].

Rectal cancer is classified according to the 8th AJCC Staging Manual for TNM classification [23]. The tumour stage depends on the extent of invasion into the layers of the rectal wall. T1 is confined to the mucosa. Further extension into the muscularis propria is regarded as T2, and extension beyond the rectal wall into the mesorectal fat is T3. Invasion into an adjacent organ, such as the prostate, uterus, or peritoneum, is classified as T4.

### 2.3. Endorectal Ultrasound Scanning Technique

ERUS examination may vary depending on preferences and the available equipment. A cleansing enema is recommended to ensure a clean rectum, thereby avoiding artifacts from residual stool. Patients should be placed in a position that allows easy access for the probe, such as the left lateral decubitus position or the lithotomy (gynaecological) position. An endocavity probe, such as a biplane, a convex array, a 360° micro convex array, an end-fire probe, or a flexible echoendoscope, with frequencies ranging from 5 to 15 MHz, may be used. Acoustic coupling is important for optimal imaging and is achievable using successive water instillation and deflation of air. A water-filled balloon covering the transducer may also be used; however, it is important that it is carefully adjusted to avoid tumour compression and false findings.

An initial digital rectal examination is performed, providing the operator with information on sphincter tonus, the direction of the anal canal, and tumour characteristics. After introducing the probe into the rectum, the ERUS examination is performed from the top of the rectum to the anal canal. T stage assessment depends on the maximal depth of tumour invasion into the rectal wall and surrounding structures.

### 2.4. Elastography—Change in Tumour Stiffness Caused by Malignant Transformation

**Figure 1:**
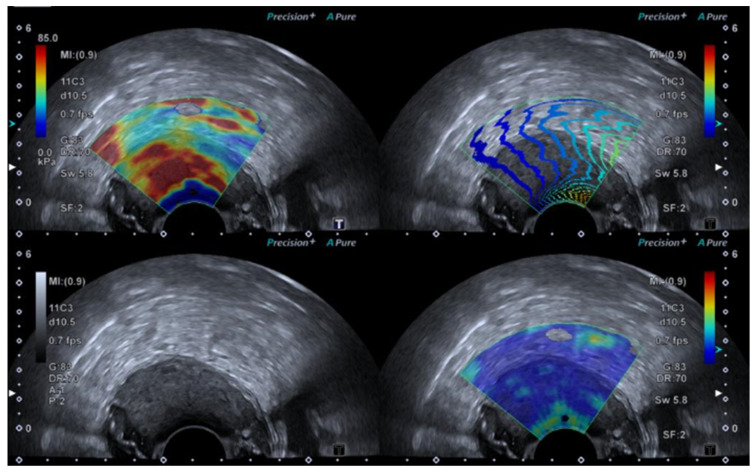
SWE ultrasound showing a t3b tumour in a 75-year-old male.

**Figure 2:**
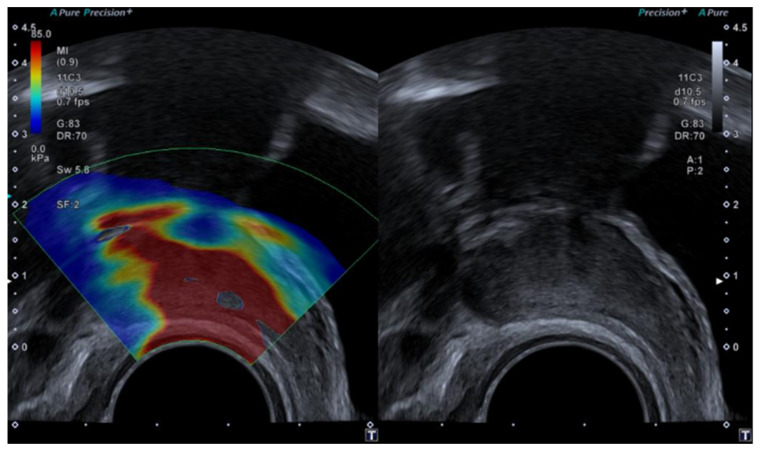
SWE ultrasound showing a t3b tumour in a 70-year-old female.

**Figure 3:**
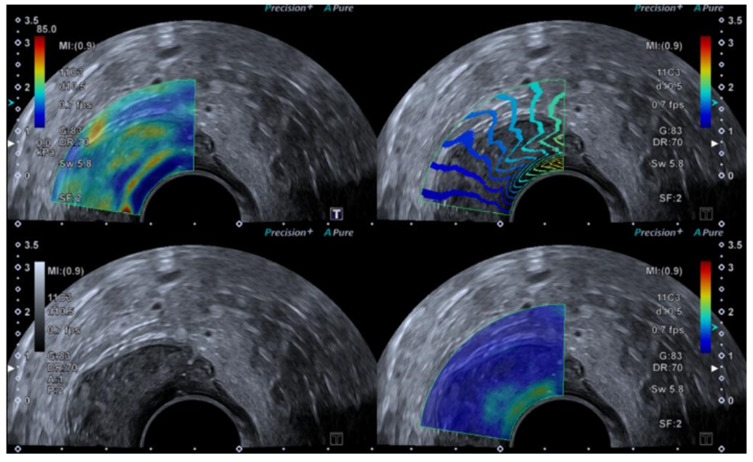
SWE ultrasound showing a polyp in an 85-year-old female.

A conventional ERUS examination is conducted to assess the tumour stage and surrounding tissue before applying elasticity imaging. The elasticity imaging is then switched on and may be performed as either SE or SWE [25] (Figure 1, Figure 2 and Figure 3).

For SE, the strain elastogram is achieved by applying pulsatile pressure either by slightly joggling the transducer or using a syringe connected to a water-filled balloon covering the transducer. When a satisfying reproducible real-time elastogram is achieved, the image is frozen. The images may be interpreted using the ratio between the lesion and reference tissue, which may include the normal rectal wall or perirectal fatty tissue, or as a continuous visual analogue score (VAS) and/or a categorical W-score [26,27,28].

SWE uses a push pulse to induce shear waves, and an elastogram is created by monitoring shear wave speed through the tissue. The transducer is held still, without applying pressure to the tumour area, for approximately 5–10 s until a satisfying elastogram is created on the monitor. A region of interest (ROI) may be placed covering the entire lesion or placed within the stiffest areas of the tumour. The elastography index is reported as either the mean or the maximal value of the ROI. SWE may also be interpreted using a visual colour scale [29,30,31,32].

Several cut-off values have been suggested for both SE and SWE (see Table 1); however, it is important to keep in mind that the elastography value differs depending on the technical approach, equipment, and software used, as well as intra- and inter-variation between operators.

## 3. Prostate

### 3.1. Introduction

Prostate cancer (PCa) is the second leading cause of cancer-related death in men. Its initial stages are asymptomatic; therefore, prostate evaluation is essential to reduce the risk of death [36,37,38,39]. A total of 70–75% of all prostate cancers originate in the peripheral zone (PZ). Despite advances in prostate imaging, a positive digital rectal examination (DRE) and/or increased prostate-specific antigen (PSA) levels remain the gold standard indicators for performing prostate gland biopsy for the diagnosis of PCa [38,39]. However, with recent advances, imaging methods, such as US-based imaging, mpMRI, mpMRI–US fusion imaging, and PET imaging, have become the primary choice for PCa detection and localisation, depending on the biological behaviour of the tumour [40,41].

Transrectal ultrasound (TRUS) is currently the preferred technique that is reliably used during diagnosis, primarily for guiding biopsies. However, it has limited sensitivity and specificity (between 40 and 50% for PCa detection) and is not significantly improved by the use of Colour/Power Doppler [42]. Transrectal ultrasound elastography appears to be a technique that is capable of mapping tissue elasticity, which could be useful in detecting and locating cancerous areas within the prostate. Two ultrasound elastography techniques have been developed based on different approaches: the first approach entails the use of the quasi-static method, and the second employs the transient shear wave technique [37].

### 3.2. Prostate Anatomy and Scanning Technique

McLaughlin (2005) and Weinred (2016) highlight that the prostate can be defined by four main zones: the peripheral zone (PZ), situated on the posterior and lateral side of the prostate; the transition zone (TZ), surrounding the prostatic urethra; the central zone (CZ), located in the base of the prostate behind the transition zone, surrounding the left and right ejaculatory ducts; and the anterior fibromuscular stroma (AFS) zone, which is a small area of tissue situated on the anterior side of the prostate [43,44]. On the other hand, Weinreb (2016) divides the prostate gland into the base, midportion, and apex, each with six sectors on each side, as described in Table 2:

For PCa classification, the system is based on the degree of glandular differentiation and the overall pattern of tumour growth under relatively low microscopic magnification, named the Gleason score (GS). Five grades of growth have been identified in order of increasing aggressiveness, and two grades have been recorded for each tumour due to the variable histology of the tumours: a primary or predominant pattern (Gleason 1_5) and a secondary or lesser pattern (Gleason 1_5). The GS is the sum of the primary and secondary patterns and ranges from 2–10. Although the GS system has been widely used for several decades, it has a few weaknesses. To overcome these weaknesses, “grade groups” (GGs) were introduced by the International Society of Urological Pathology (ISUP) and are defined as follows: GG1 (GS _ 6), GG2 (GS 3+ 4 = 7), GG3 (GS 4 + 3 = 7), GG4 (GS 8), and GG5 (GS 9_10). GGs are important parameters for therapeutic decision making for PCa patients [45].

Recently, clinically significant prostate cancer, defined by at least one biopsy core with a Gleason score of 3 + 4 or 6 with a maximum cancer core length greater than 4 mm, is considered to be associated with cancer progression [46].

### 3.3. Elastography in Prostate Lesions

Elastography has improved prostate examination as a diagnostic method by measuring and visualising prostate stiffness using a colour map, allowing for an accurate assessment of the size, position, and stiffness of suspected PCa lesions [39]. Strain elastography (SE) and shear wave elastography (SWE) appear to be promising in the diagnosis of prostate cancer. Although there are few studies in this area comparing the two techniques, recent studies have demonstrated that SWE is useful for the detection of PCa, showing that tissue stiffness is regarded as a strong indicator of malignancy [39,41]. Regions of increased stiffness have been translated into the prostate score. Elastography measures are shown in Table 3.

**Figure 4:**
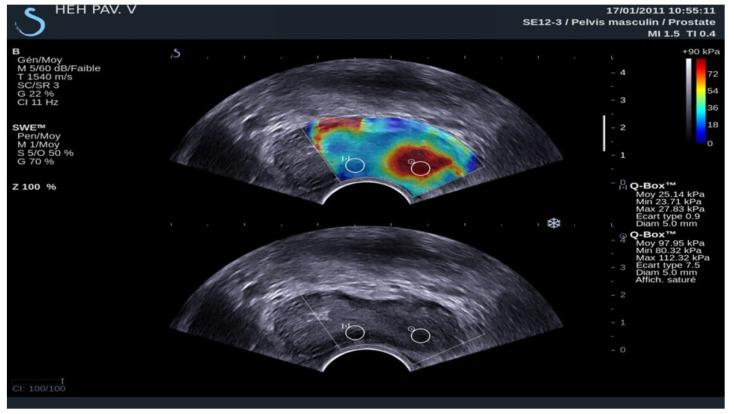
A 65-year-old patient with elevated PSA without other clinical data of interest. Image obtained using a SuperSonic Aixplorer ultrasound scanner with a multifrequency transrectal probe, bandwidth 3–12 MHz. Prostate cancer: the image identifies that the ROI of the left lobe has a mean hardness of 97.95 kPa, which is highly suspicious of malignancy. This finding was subsequently confirmed by biopsy.

**Figure 5:**
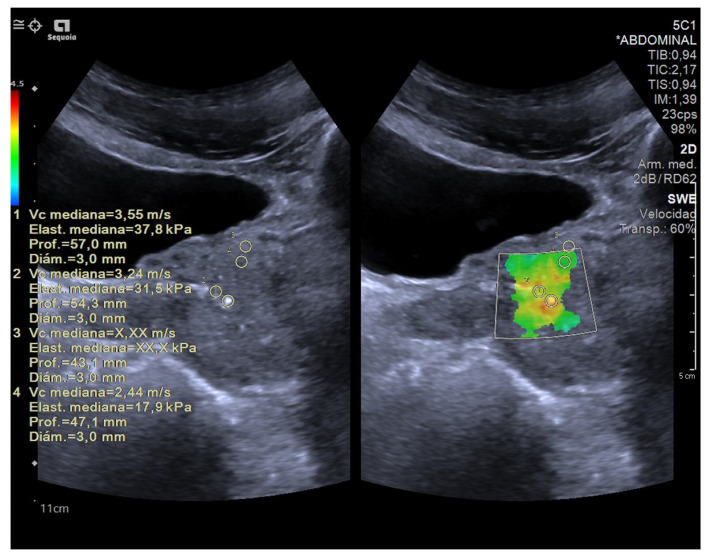
41 year-old patient with elevated PSA without other clinical data of interest. Prostate ultrasound image with a suprapubic approach performed using a SIEMENS Sequoia ultrasound device with a Convex 5C1 probe. The elasticities of the tissues observed in the ROIs were 37.8 kPa in ROI 1; 31.5 kPa in ROI 2 and 17.9 kPa in ROI 4; ruling out the absence of malignant disease. Adjacent to the prostate, a hypoechoic area was identified, corresponding to an abscess, with the diagnosis being prostatitis. The absence of data indicating tissue stiffness in ROI 3 may be due to the heterogeneity of the sample.

SE was interpreted based on the colour of the area in the elastography map. On the other hand, SWE was interpreted using a cut-off value in kilopascals. The SWE stiffness values in our study were based on transverse (axial) plane imaging (Figure 4 and Figure 5).

According to the World Federation for Ultrasound in Medicine and Biology guidelines, for SWE, stiffness values >35 kPa (Young’s modulus) are interpreted as being suggestive of prostate cancer [39]. Recently, prostate biopsy guided with elastosonography has become an alternative to conventional ultrasound-guided systematic biopsy, showing a 50% reduction in the number of biopsy cores required using elastography [38].

## 4. Scrotum

### 4.1. Introduction

The incidence of testicular cancer is expected to increase worldwide in the future [48]. Ultrasound (US) B-mode and doppler US are the gold standard methods of detection and are typically the first line of imaging examinations in patients with a palpable testicular lesion because of their low cost, accessibility, the location of the testicles, and their diagnostic accuracy. US can visualise and characterise testicular lesions but not alway differentiate between benign and malignant lesions [32,33,49,50]. The term “focal lesion” describes a nodular structure differing from the surrounding testicular tissue. Multiparametric US includes both magnetic resonance imaging (MRI) and contrast-enhanced US [50]. MRI has also proven to be useful and accurate in visualising and characterising testicular lesions [51,52,53,54,55]; however, it is typically used as a secondary add-on imaging tool.

In recent years, strain elastography (SE) and shear wave elastography (SWE) of the testicles have become standard practice in many imaging departments, as most ultrasound equipment now includes an elastography module as standard, although its clinical use is still limited. A typical focal lesion will differ in tissue heterogeneity compared with the surrounding testicle parenchyma. Therefore, elastography is a useful tool for differentiating benign from malignant tissue. Elastography of the scrotum is easily and rapidly performed without any additional discomfort to the patient. Both SE and SWE have been used in the assessment of testicular lesions [49,51,56,57,58,59,60,61,62]. SE elastography aims to assess tissue stiffness by quantifying tissue deformation, whereas SWE measures the speed of propagation when applying force [17,63].

### 4.2. Scrotum Anatomy and the Scanning Technique

**Figure 6:**
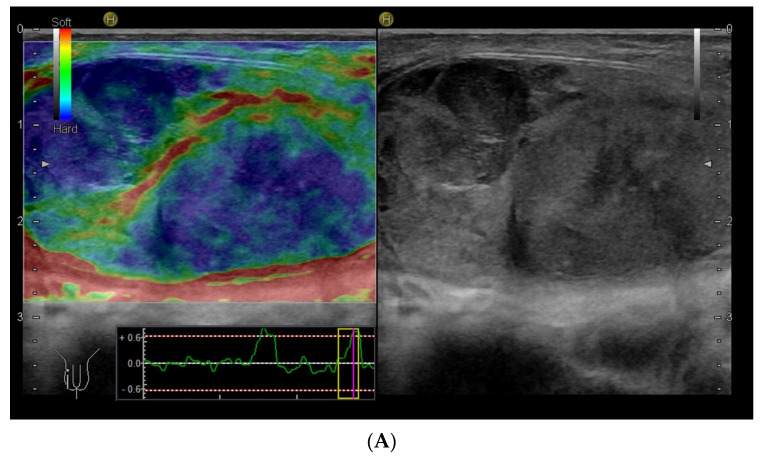

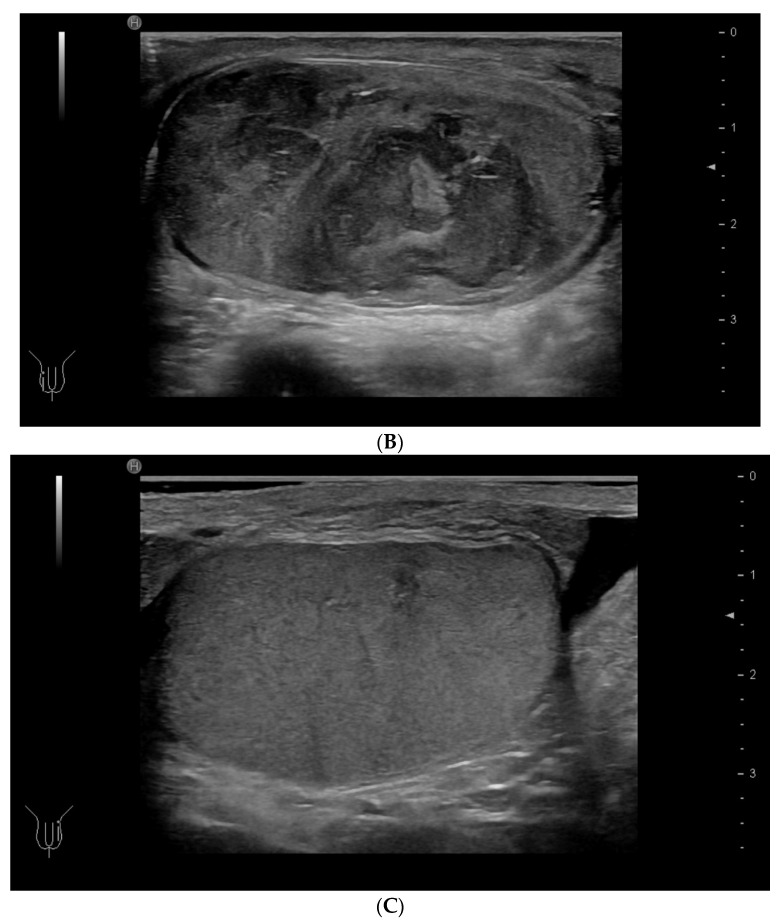
Ultrasound images of a 39-year-old male. The indication for ultrasound investigation was suspected orchitis or malignancy. The patient experienced pain in the right testicle for more than 2 days. The patient was seen in the emergency room for assessment of epididymitis and started treatment with ciprofloxacin. There was no improvement with treatment, and the right testicle became more tender and swollen. The ultrasound showed no signs of epididymitis (**A**). The right testicle (left side of the figure shows the elastogram, and B-mode is seen on the right side). The testicle revealed a right-sided tumour, which appeared highly suspicious using elastography (**B**). The B-mode image, showing a tumour (**C**). B-mode image of the left testicle, showing normal tissue (The pathology showed a seminoma testicular tumour with 2% of choriocarcinoma. The patient underwent surgery for removal of the right testis.

Within the scrotum, the two testicles are divided by a septum. Each testicle is oval-shaped and typically measures 4 cm in length, 3 cm in width, and 2 cm in height [64]. The anatomy of the testicles can be seen in Figure 6C, and an ultrasound image including SE is shown in Figure 6A,B. A high-frequency linear transducer is typically the preferred and best choice, but in cases with an enlarged scrotum, a curved array transducer may be a better choice.

Patients are placed supine, and the scrotum is elevated, with the penis positioned along the patient’s abdominal wall, covered by the patient’s hand and preferably also a sheet for privacy. The testicles are evaluated using B-mode in both longitudinal and transverse planes. Individual adjustment is performed for every scan, including depth and grayscale gain. Testicular size, placement, and echogenicity are evaluated individually and compared to the other testicle.

### 4.3. Elastography in Testicular Lesions

After the B-mode examination, elastography is performed by placing the transducer using the B-mode findings to locate the point of interest in the testicle. The transducer is in direct contact with the skin and is held still during measurement. The SWE elastography mode will typically be labelled at the bottom and displayed next to the keyboard. After activation of the elastography module, a region of interest box will appear, and it should be placed above the area of interest. It is recommended that more than one measurement is performed. Also, it may be recommended to perform elastography measurements in the contralateral testicle.

In SE elastography, the testicular lesion is vertically compressed using light pressure, which is performed by the operator using the transducer, until a visual scale is displayed on the screen using a colour-coded map called an elastogram. The elastogram is placed on over of the grayscale B-mode. Repetition is recommended. To calculate a strain ratio (SR), an ROI is drawn by the operator in the reference tissue and in the target lesion. SE testicular 6-point elasticity scores have been presented [65,66,67], whereas others have used Itoh 5-point scale for breast lesions [67]. The score has been adapted to characterise testicular lesions (Table 4).

Furthermore, elastography in the testicles is performed not only in testicular lesions but also in other testicular conditions, such as orchitis, fibrosis, abscess, hydrocele, microlithiasis hematoma, infarction, fertility, and torsion [66,68,69,70,71,72]. However, this was outside the scope of this study. Table 5 shows an overview of elastography in testicular lesions.

## 5. Discussion and Conclusions

Elastography is a technique that evaluates organ tissue either in a colour-coded map or through an objective quantitative value. Most ultrasound machines include elastography as a standard tool, providing high diagnostic accuracy when a multi-US imaging approach is used to evaluate the tissue.

The recent diagnostic advancement of elastography into clinical practice is evident in many departments. Given that most ultrasound machines include an elastography module, more and more clinicians are gaining interest in using elastography. Therefore, it is important to provide knowledge about elastography values in benign and malignant tissue.

In prostate cancer, ultrasound elastography has been shown to be a good method for diagnosing malignant lesions; however, few studies have compared both methods. SWE has been found to be useful for detecting prostate cancer, with higher sensitivity and specificity. The Gleason score was used in all studies to classify glandular differentiation and tumour growth, making it easier to understand the impact of this technique on PCa detection. The use of different equipment could influence the results.

In testicular cancer, all the studies reported tumours as stiffer than the surrounding benign tissue. We saw a large spread in both SR and SWE values in the studies. Although elastography shows promise in differentiating malignant from benign testicular tissue, the clinical utility appears limited due to variation in the reported values and the lack of a cut-off value for malignancy. The variation in kPa and SR values may also partly be caused by variation in the ultrasound machines, the quality of the machines, and the frequency range of the probes used in the included studies. The number of patients with testicular lesions was also limited. Therefore, large cohort studies are needed to establish cut-off values in the assessment of testicular lesions.

In conclusion, elastography is widely used for imaging the male pelvic region. SE and SWE offer different types of evaluations that are of value for diagnostic evaluation. SE is more operator-dependent due to the requirement for active movement of the transducer to produce compression. However, both techniques can be easily learned with little training. Performing SE or SWE as part of a standard US examination of male pelvic structures should be considered. Elastography in malignant lesions has been widely used, and it is widely accepted among authors that elastography displays higher tissue stiffness accuracy. However, there is an overlap in stiffness in soft tissue, making it a challenge to provide cut-off elastography values.

## Figures and Tables

**Table 1:** Overview of elastography in investigating malignant rectal lesions.

Study, Year	Lesion	n	SE	SWE	Mean Elastography	Maximum Elastography	Cut-Off Values
Waage, 2011 [33]	Malignant/benign	68	X		T0: 0.55 (SD 0.42) T1–4: 9.26 (SD 12.28)		>1.25
Waage, 2015 [34]	Malignant/benign	115	X		T0: 1.03 (0.88–1.22) T1–4: 5.53 (4.27–6.79)		>1.25
Waage, 2015 [35]	ERC/benign	59	X		T0: 1.03 (0.88–1.22) T1: 2.44 (1.67–3.33) T2: 6.29 (2.30–11.55) T3–4: 4.52 (2.92–11.01)		T0 < 0.80 T1–2 0.81–1.10 T3–4 > 1.11
Xiao, 2018 [29]	Malignant (T3–4)	53	X		NR	Visual colour scale	NR
Oien, 2019 [27]	ERC/benign	96	X		NR		NR
Chen, 2017 [29]	Malignant/benign	100		X	NR		T0 < 26.9 kPa T1 ≥ 26.9 kPa T2 ≥ 70.3 kPa T3 ≥ 112.0 kPa
Fan, 2019 [31]	Malignant	55		X	T1: 15.9 ± 7.5 (5.8–31.3) T2: 40.4 ± 20.3 (16.5–83.7) T3–4: 51.2 ± 15.6 (19.6–75.9)	T1: 29.6 ± 11.2 (10.9–53.2) T2: 76.7 ± 33.1 (23.9–126.3) T3–4: 104.8 ± 20.5 (65.1–137.1)	NR
Li, 2019 [30]	Malignant/benign	96		X	T0: 35.2 ± 24.7 T1–4: 89.7 ± 29.5	T0: 43.7 ± 31.6 T1–4: 103.2 ± 36.2	Emean 61.3 kPa Emax 63.4 kPa
Loft, 2022 [32]	ERC/benign	86		X	T0: 24.87 ± 11.92 (21.86–27.88) T1: 40.99 ± 17.63 (30.34–51.56) T2: 72.12 ± 25.91 (57.84–86.41)		>39.9 kPa

n, number of patients; SE, strain elastography; SWE, shear wave elastography; ERC, early rectal cancer (i.e., T1–2); NR, not reported.

**Table 2:** Division of the prostate gland [44].

Division of Prostate Gland	Sectors
Base	AS: anterior fibromuscular stroma TZ: anterior and posterior transition zone PZ: anterior and posterior zone CZ: central zone around the ejaculatory ducts
Midportion	AS: anterior fibromuscular stroma TZ: anterior and posterior transition zone PZ: anterior, posteromedial, and posterolateral peripheral zone
Apex	AS: anterior fibromuscular stroma TZ: anterior and posterior transition zone PZ: anterior, posteromedial, and posterolateral peripheral zone

**Table 3:** Overview of elastography in investigating malignant prostate lesions.

Study, Year	Probe Mhz	Participants/Tumours (n)	Type: Malignant (M); Benign (B)	Gleason Scores			Sensitivity/Specificity
				N	SE	SWE (Pixel-/Region-Wise)	
Secasan, 2022 [38]	endocavitary	356/223	M: 223 B: 133	G1: 90 G2: 14 G3: 61 G4:31 G5:27	-	G6	NR
Wildeboer, 2019 [41]		48	M: 48	G6(3 + 3): 1 (2.1%) G7(3 + 4): 30 (62.5%) G7(4 + 3): 6 (12.5%) G7(>4 + 3): 11 (22.9%)	-	PW ≥G6: 0.62 >G3 + 4 = 7:0.67 RW ≥G3 + 3 = 6:0.62 >G3 + 4 = 7:0.73	NR
Tyloch, 2022 [39]		434(SE) 453(SWE)	M:434(SE), 453(SWE) B: 611(SE), 552(SWE)	G6(3 + 3): 3 G7(3 + 4):19 G7(4 + 3): 6 G9(4 + 5):2	-	B: 36.43 G3: 43.41 G4: 55.93 G5: 66.81	SE: G3: 52.7; G4: 70.5; G5: 55.6/{71.8} SWE: G3: 54.6; G4: 81.6; G5: 93.8/{70.2}
					SE and SWE (overall stiffness cut-off value = 35 kPa		
Dai, 2021 [45]		221/85	M: 97 B: 115	G1: 3 G2: 14 G3: 9 G4: 10 G5: 13	-	G1/G2: 17 (65.06) G3-G5: 32 (88.65)	High GG 81.3%/82.6%—cut-off value for EMax > 84 kPa 78.1%/76.5%, cut-off value for EMean > 71 kPa 68.8%/70.6% cut-off value for EMin > 60 kPa 46.9%/94.1% cut-off value for SD > 8.3 kPa
Wei, 2021 [47]		212/212	M: 212	G ≤ 6: 42 (19.8%) G7(3 + 4): 75 (35.4%) G7(4 + 3): 39 (18.4%) G > 7: 56 (26.4%)	-	G ≤ 7(3 + 4):28 (154.2) G ≥ 7(4 + 3): 89 (134.3)	NR

G: Gleason Score; PW: pixel-wise; RW: region-wise; SE: strain elastography; SWE: shear wave elastography.

**Table 4:** Overview of the 5- and 6-point elastography scores.

Elastography Score (ES)	Score 1	Score 2	Score 3	Score 4	Score 5	Score 6
6-point (65,66)	Benign	Benign	Benign	Malignant	Malignant	Malignant
	Lesion is completely green (red elastography score), with some red spots	Entire lesion is evenly shaded green	Lesion is almost completely green with some small blue spots	Lesion is green at the periphery with central blue area	Lesion is almost completely blue with central small green/red areas	The lesion is completely blue (hardest)
5-point (67)	Benign	Benign	Benign	Malignant	Malignant	
	ES is the same as the surrounding tissue	Lesions are mainly soft compared to normal tissue	The periphery of a lesion with no strain at the centre	Harder nodule than the adjacent tissue and indicates no strain in the entire lesion	No strain in the entire lesion and the surrounding area, indication infiltration of cancer cells	

**Table 5:** Overview of elastography in investigating malignant testicular lesions. All studies included B-mode scans.

Study, Year	Probe MHz	Tumours (n)	Lesion Type	RTE/SE	SR	SWE	Sensitivity/Specificity
Pallwein, 2006 [73]	NR, 14	15	Malignant	15 hard lesions	-	-	NR
Grasso, 2010 [74]	Linear, 7.5	2	Malignant	2 hard lesions	-	-	NR
Aigner, 2012 [75]	Linear, 14	34	Malignant	34 hard lesions	-	-	100%/81%
Goddi, 2012 [57]	Linear, 5–14/6–14	38	Malignant	87.5% were hard (ES 4–5) *	-	-	87.5%/98.2%
Marsaud, 2014 [76]	Linear, 9–14	26	Malignant	NR	-	-	NR
Pastore, 2014 [77]	Linear, 5–14	30	Malignant	-	2.1–56	-	NR
Trottmann, 2014 [78]	Linear, 9	15	Malignant	-	-	2.42/1.94 m/s	NR
Dikici, 2016 [62]	Linear, 4–15	15	Malignant	-	-	10.6–47 kPa ^	NR
Schroder, 2016 [79]	Linear, 5–17	46	Malignant	-	0.5–3.9	-	NR
Pozza, 2016 [49]	Linear, 7–15	37	Malignant	30 = ES3, 7 = ES2 *	-	-	81.8%/79.1%
Pedersen, 2017 [51]	Linear, 9	19	Malignant	-	-	mean 1.92 m/s	NR
Auer, 2017 [80]	Linear, 6–15/5–13	12	Malignant	12 hard lesions	-	-	100%/72.1%
Pedersen, 2018 [81]	Linear, 4–9	23	Malignant	-	-	mean 2.09 m/s	NR
Konstantatou, 2019 [82]	Linear, 14–6	31	Malignant	-	median 4.64	-	68.8%/81.6%
Reginelli, 2019 [83]	Linear, 5–13	46	Malignant	40 hard, 6 soft–medium	-	-	NR*
Roy, 2020 [68]	Linear, 10	64	Malignant	-	-	mean 23.1 kPa	82%/81%
Bhansali, 2022 [59]	Linear, NR	1	Malignant	1 ES5 *	8.2	-	NR
Ladke, 2022 [84]	Linear, NR	1	Malignant	-	-	50 kPa/1.6 m/s	NR
Corcioni, 2022 [61]	Linear, 10–15/7–14	15	Malignant	9 hard, 6 intermediate	-	-	NR

NR = not reported; SR = strain ratio; RTE = real-time elastography; * Itoh elasticity score used; ^ mean seminoma/mean non-seminoma. NR* = combined use of US, Doppler, and RTE (with a sensitivity of 100% and a specificity of 83%).

## Data Availability

This study used different articles available on internet; the images were providing by colleagues.
